# Comprehensive analysis: Necroptosis-related lncRNAs can effectively predict the prognosis of glioma patients

**DOI:** 10.3389/fonc.2022.929233

**Published:** 2022-08-10

**Authors:** Desheng Chen, Chao Dou, Haiyu Liu, Binshun Xu, Bowen Hu, Liangwen Kuang, Jiawei Yao, Yan Zhao, Shan Yu, Yang Li, Fuqing Wang, Mian Guo

**Affiliations:** ^1^ Department of Neurosurgery, The Second Affiliated Hospital of Harbin Medical University, Heilongjiang, China; ^2^ Department of Pathology, The Second Affiliated Hospital of Harbin Medical University, Heilongjiang, China; ^3^ China Pharmaceutical Enterprises Association, Heilongjiang, China

**Keywords:** gliomas, immune, TEM, biomarker, necroptosis-related lncRNAs

## Abstract

Glioma is the most common and fatal primary brain tumor in humans. A significant role for long non-coding RNA (lncRNA) in glioma is the regulation of gene expression and chromatin recombination, and immunotherapy is a promising cancer treatment. Therefore, it is necessary to identify necroptosis-related lncRNAs in glioma. In this study, we collected and evaluated the RNA-sequencing (RNA-seq) data from The Cancer Genome Atlas (TCGA, https://www.ncbi.nlm.nih.gov/, Data Release 32.0, March 29, 2022) glioma patients, and necroptosis-related lncRNAs were screened. Cox regression and least absolute shrinkage and selection operator (LASSO) analysis were performed to construct a risk score formula to explore the different overall survival between high- and low-risk groups in TCGA. Gene Ontology (GO) and pathway enrichment analysis (Kyoto Encyclopedia of Genes and Genomes (KEGG)) were performed to identify the function of screened genes. The immune correlation analysis showed that various immune cells and pathways positively associated with a patient’s risk score. Furthermore, the analysis of the tumor microenvironment indicated many immune cells and stromal cells in the tumor microenvironment of glioma patients. Six necroptosis-related lncRNAs were concerned to be involved in survival and adopted to construct the risk score formula. The results showed that patients with high-risk scores held poor survival in TCGA. Compared with current clinical data, the area under the curve (AUC) of different years suggested that the formula had better predictive power. We verified that necroptosis-related lncRNAs play a significant role in the occurrence and development of glioma, and the constructed risk model can reasonably predict the prognosis of glioma. The results of these studies added some valuable guidance to understanding glioma pathogenesis and treatment, and these necroptosis-related lncRNAs may be used as biomarkers and therapeutic targets for glioma prevention.

## Introduction

Glioma is a common malignant tumor of the central nervous system, and the prognosis is inferior even after years of treatment (1). The main clinical treatments for gliomas are currently surgery, radiotherapy, and chemotherapy, but they usually fail to achieve the desired results (2, 3). Therefore, further studying the clinical diagnosis and treatment methods of glioma, exploring new risk factors and molecular markers, and developing new therapeutic targets that have improved the clinical prognosis of patients with glioma are necessary.

Nowadays, many mechanisms and possible therapeutic pathways have been investigated. For example, Mian Guo et al. verified that cyclophilin maintains glioma-initiating cell stemness by regulating Wnt/β-catenin signaling and TUSC3 suppresses glioblastoma development by inhibiting Akt signaling (4, 5). Zhang et al. verified reciprocal control of ADAM17/EGFR/Akt signaling and miR-145 drives glioblastoma multiforme (GBM) invasiveness (6). These conclusions are of great guiding significance to clinical research. However, these studies have not significantly improved clinical progress.

As we all know, long non-coding RNAs (lncRNAs) are transcripts of more than 200 nucleotides that are not translated into proteins. These transcripts include intergenic transcripts, enhancer RNAs (eRNAs), and sense or antisense transcripts that overlap with other genes (7). LncRNAs have been proposed to function in various ways, including transcriptional regulation, the organization of nuclear domains, and the regulation of proteins and RNA molecules (8). Moreover, several studies have shown that necroptosis has two effects on cancer: firstly, the key regulators of necroptosis contribute to the progression and metastasis of cancer alone or together; alternatively, necroptosis can also act as an ‘insurance’, preventing tumor growth and metastasis when the cell’s apoptosis is disrupted. Therefore, is it more worthwhile to study the possible mechanisms of necroptosis-related long non-coding RNA.

Meanwhile, patients with glioma may benefit more from immunotherapy due to its safety and fewer side effects. The treatment provides new insights into clinical treatment management. Since few patients could benefit from immunotherapy, the overall disease control rate and treatment strategies still need improvement. The imbalance of the immune system has a vital role in the development of glioma characterized by an immunosuppressive disease. Thus, the regulatory mechanism of the tumor immune microenvironment (TIME) should be further explored to determine effective biomarkers that precisely predict prognosis and considerably optimize personalized immunotherapy management.

Recently, we found an article that necroptosis-related long non-coding RNA is associated with immune infiltration of colon cancer in the *Front Mol Biosci*. A risk scoring system was constructed to predict the prognosis of patients (9). Whether glioma has a similar mechanism requires further research.

**Figure 1:**
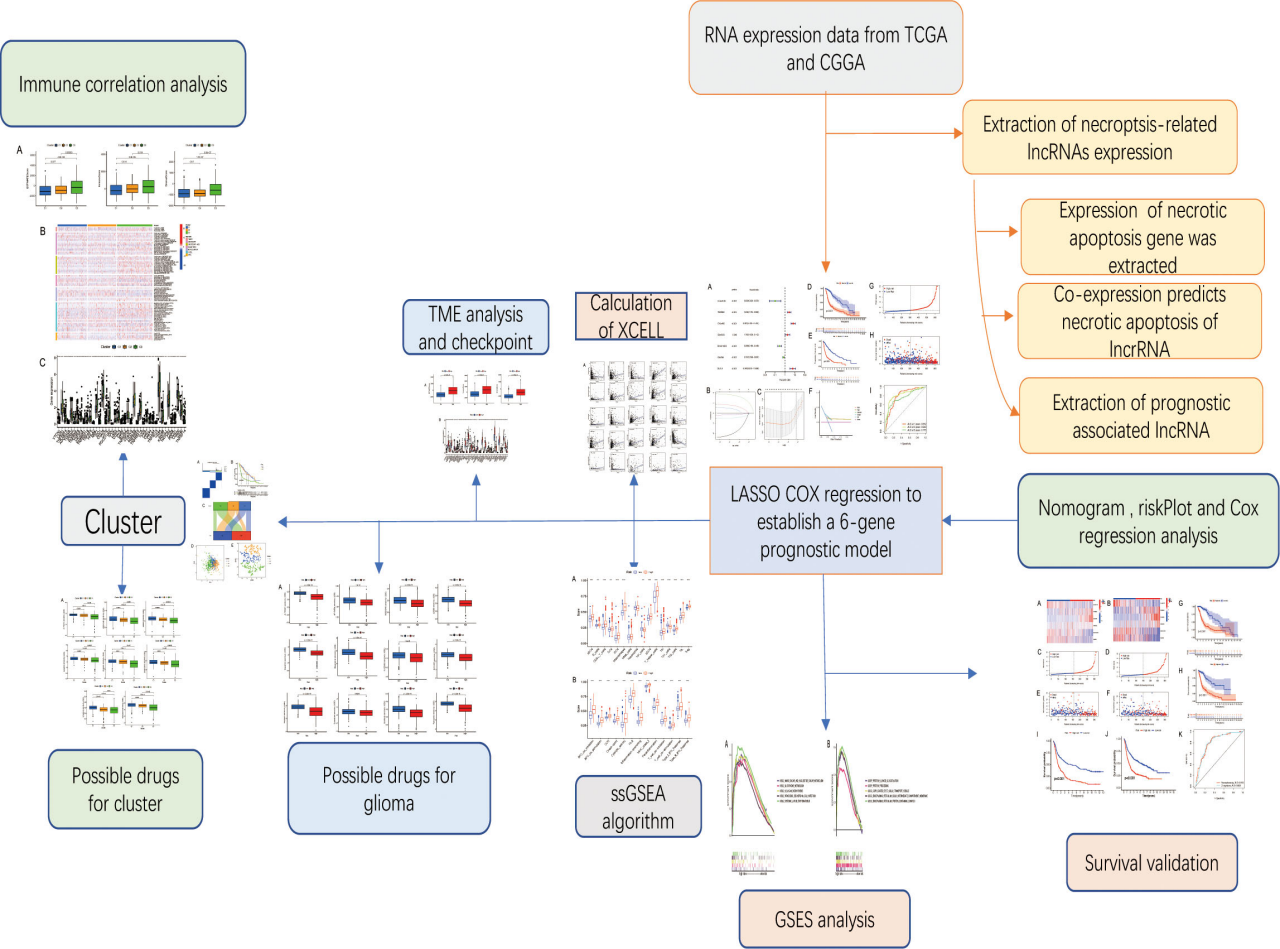
Workflow diagram. The specific workflow graph of this work.

To use the scoring system above, glioma’s activity level during this process was measured. Based on the glioma cases in The Cancer Genome Atlas (TCGA) databases (https://portal.gdc.cancer.gov/), we calculated this score, investigated its clinical connection, and discussed the possible mechanism behind it, providing theoretical guidance for the subsequent clinical treatment based on the molecular mechanism of necroptosis-related long non-coding RNA (10). The workflow is shown in **Figure 1**.

## Methods

### Study cohort

We obtained the RNA-sequencing (RNA-seq) data for human glioma (GBM and lower-grade glioma (LGG)) from TCGA and Chinese Glioma Genome Atlas (CGGA) and the corresponding clinical information, including age, gender, and survival information.

Sample exclusion criteria were follow-up time was less than 30 days, grade unknown, death unknown, age unknown, etc.

### Acquisition of glioma data and data processing

TCGA database (https://portal.gdc.cancer.gov/) was used for collecting RNA-seq data of 698 LGG and GBM samples and five normal subjects, as well as relevant clinical information. The RNA-seq and clinical data of CGGA325 and CGGA693 were collected from the CGGA (http://www.cgga.org.cn). Perl software was used for data processing to transform ensemble-ID into gene names and access to clinical information.

### Co-expression analysis to extract necroptosis-related lncRNAs

Sixty-seven necroptosis genes were downloaded from GSEA-MSigDB (http://www.gsea-msigdb.org/gsea/login.jsp). Limma is an R/Bioconductor software package that provides an integrated solution for analyzing data from gene expression experiments. It is used to conduct background expression value correction and data normalization (11). In this study, the expression of necroptosis-related genes (NRGexp) was extracted using the ‘limma’ package. Then, to obtain necroptosis-related lncRNAs (NRlncRNAs), a co-expression network was performed, and it was visualized by (‘graph’) packages with corFilter = 0.1 and pvalueFilter = 0.001. Differential expression analysis was performed between tumor and normal samples and visualized by (‘heatmap’) packages with logFCfilter = 1 and fdrFilter = 0.05.

### Risk model construction and verification

Cox regression analysis was used in evaluating the prognosis value of the NRlncRNAs. The hazard ratio (HR), 95% confidence interval (CI), and *p*-values of each variable are displayed on the forest map. In this study, HR was used to determine protective NRlncRNAs (HR < 1) and risk NRlncRNAs (HR > 1). With the ‘glmnet’ R package, the least absolute shrinkage and selection operator (LASSO) regression model was constructed to screen optimal NRlncRNAs for prognosis. The optimal value of the penalty parameter (λ) was determined based on the results of 1,000 cross-validation runs. The weighted regression coefficients and expression levels of prognostic NRlncRNAs were derived, and the following formula was used to calculate each patient’s risk score of survival: Risk score (RS) = ∑Ni − 1Expi * Coie . Patients with glioma in TCGA were divided into low- and high-risk groups based on their median risk score. The Kaplan–Meier analysis was used to compare the survival times of the two groups. Receiver operating characteristic (ROC) analyses were performed using the ‘survival’, ‘survminer’, and ‘timeROC’ packages for evaluating the prediction accuracy of different genes and risk scores. Progression-free survival (PFS) analysis was performed on the two groups to verify whether risk scores were related to the PFS of patients. Decision curve analysis (DCA) was conducted by (‘ggDCA’) package to determine the clinical benefit by quantifying the net benefits along with the increase in threshold probabilities. ROC curves were drawn separately for the dataset using the ‘timeROC’ package. The area under the ROC curve was selected, and it was compared to the performance of the survival-related lncRNAs.

### Internal verification and external verification

The samples were randomly divided into the training group and testing group at a ratio of 1:1 using the ‘caret’ R package. Then the training group was used to construct a prognostic model and the testing group to perform internal validation. Then, survival analysis was performed using the CGGA dataset (CGGA325 and CGGA693). Meanwhile, the ROC curve was constructed to compare with the model constructed by the predecessors (12).

### Univariate Cox and multivariate Cox regression analyses

To assess whether risk score can be regarded as an independent predictor of overall survival (OS) of glioma patients, univariate Cox and multivariate Cox regression analyses were performed with a risk score, gender, age, and grade as variables using the R ‘survival’ package.

### Nomogram and survival analysis of different clinical features

The ‘rms’ package created a nomogram for predicting glioma patients’ survival. We used the ‘limma’ and ‘ggpubr’ packages to perform expression and survival analyses of various clinicopathological characteristics. The validity of the model can be verified through this analysis.

### Functional annotation of necroptosis-related lncRNAs

Gene Ontology (GO) pathway enrichment analysis and Kyoto Encyclopedia of Genes and Genomes (KEGG) functional enrichment analysis were performed using the R package ‘clusterProfiler’ to verify the biological functions of the necroptosis-related lncRNAs.

### Immune correlation analysis of necroptosis-related lncRNAs

The relationship between risk scores and immune cells was predicted based on the CIBERSORT algorithm. ‘Limma’, ‘survival’, and ‘survminer’ packages were used to determine the survival differences between high- and low-score patients in each immune cell type.

### Gene set variation analysis

For an in-depth analysis of the biological processes associated with the immune cell or immune-related functional pathways, the ‘GSVA’ R package for gene set variation analysis (GSVA) enrichment analysis was utilized. The single-sample gene set enrichment analysis (GSEA) (ssGSEA) was used to evaluate the infiltration level of immune cells. The infiltration score of each immune cell was calculated through ssGSEA of the ‘GSVA’ R package. The ssGSEA score was normalized to a uniform distribution, for which the ssGSEA score is distributed between 0 and 1. Moreover, the ‘limma’ package in R was also applied to display distinctions in pathway activation between low- and high-risk groups.

### Analysis of differences in tumor microenvironment and immune checkpoint

ESTIMATE was designed to count scores for reflecting the infiltration levels of immune cells and stromal cells within the tumor microenvironment (TME) based on the specific genes’ expression level of immune and stromal cells using the R package ‘ESTIMATE’ (13). The ESTIMATE algorithm was used based on the expression level of each sample to count the immune score (positively reflecting the abundance of immune cells), stromal score (positively reflecting the abundance of stromal cells), and ESTIMATE score (positively reflecting non-tumor composites). Then, the differences in scores between the high- and low-risk groups were compared and visualized using the R package ‘ggpubr’. Immune checkpoints were identified as differentially expressed in high- and low-risk groups. The variations in expression between high-risk and low-risk groups and the disparities in survival between high-risk and low-risk groups having distinct immune pathways were investigated.

### Possible drugs for glioma

This study aims to predict chemical compounds that could be used to treat patients who are in the high-risk group and low-risk group. Based on the Genomics of Drug Sensitivity in Cancer (GDSC) website, the IC50 values for the compounds were calculated. Compounds that might be used as glioma therapy have been predicted using the ‘limma’, ‘pRRophetic’, ‘ggpub’, and ‘ggplot2’ packages.

### Sample subcomponent type and survival analysis

The necroptosis-related lncRNA gene set described above was used for subtype glioma patients. Consistency analysis by using the ConsensusClusterPlus R package (v1.54.0), the maximum number of clusters is 6, and 80% of the total sample is drawn 100 times, clusterAlg = ‘hc’, innerLinkage = ‘ward.D2’. With the use of the Kaplan–Meier survival analysis of the different groups of samples from TCGA dataset, a comparison among different groups was made by log-rank test. HR (95% CI) and the median survival time (LT50) for different groups were determined. Meanwhile, the association between tumor subtypes and high-risk groups was also validated. The ‘Rtsne’ and ‘ggplot2’ packages were used to visualize data using principal component analysis (PCA) and t-distributed stochastic neighbor embedding (t-SNE).

### Cluster immune correlation analysis and drug prediction

Microenvironment analysis, immune correlation analysis, immune checkpoint identification, and potential drug prediction for tumor subtypes were performed based on the same method.

### Statistical analysis

R software (version 4.1.0) was used for statistical analysis and outcome display. For comparison between the two groups, Student’s t-test was used. Chi-square tests were used to contrast the classification variables in the training and testing tests. With the use of Pearson’s correlation test, the relationship between subtypes, clinicopathological factors, risk scores, immune check inhibitors, and levels of immune infiltration was evaluated. Survival analysis was performed using the Kaplan–Meier method with a two-sided log-rank test. All *p* < 0.05 was considered to indicate significance, and * if *p* < 0.05, ** if *p* < 0.01, and *** if *p* < 0.001.

## Result

### Extract prognosis-related necroptosis-related lncRNAs

**Figure 2:**
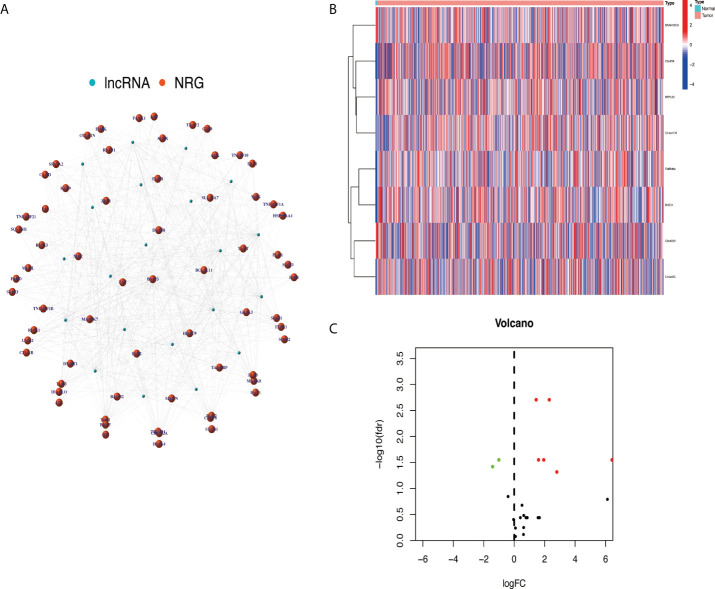
Extracted necroptosis-related lncRNAs (NRlncRNAs). **(A)** The co-expression network between lncRNA and NRG. **(B, C)** NRlncRNA heat map and volcano map. lncRNAs, long non-coding RNAs; NRG, necroptosis-related gene.

After screening, a total of 514 transcriptome samples were included in the study. A total of 67 necroptosis-related genes were extracted using the ‘limma’ package based on gene.txt downloaded from the GSEA database. Through co-expression analysis, 22 necroptosis-related lncRNAs were screened out. Finally, eight prognosis necroptosis-related lncRNAs were analyzed with differential expression analysis with the clinical samples (**Figures 2A–C**).

### Construction and validation of a risk model according to necroptosis-related lncRNAs in glioma

**Figure 3:**
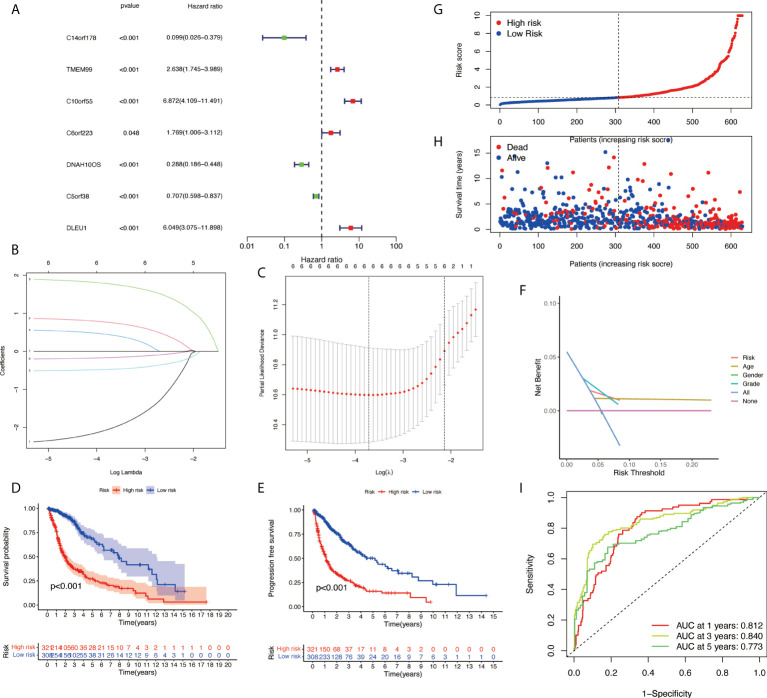
Development of necroptosis-related lncRNA (NRlncRNA) prognosis signature. **(A)** Univariate Cox regression analysis of glioma for each prognostic NRlncRNA. **(B)** Distribution of LASSO coefficient for prognostic NRlncRNAs. **(C)** Partial likelihood deviance of the LASSO coefficient distribution. **(D)** Overall survival curves for glioma patients in the high-/low‐risk group. **(E)** PFS curves for glioma patients in the high-/low‐risk group. **(F)** DCA curves for glioma patients based on clinical information. **(G)** Distribution of GBM patients based on the risk score. **(H)** Distribution of GBM patients based on survival status. **(I)** ROC curves of measuring the predictive value. LASSO, least absolute shrinkage and selection operator; PFS, progression-free survival; DCA, decision curve analysis; GBM, glioblastoma multiforme; ROC, receiver operating characteristic.

**Figure 4:**
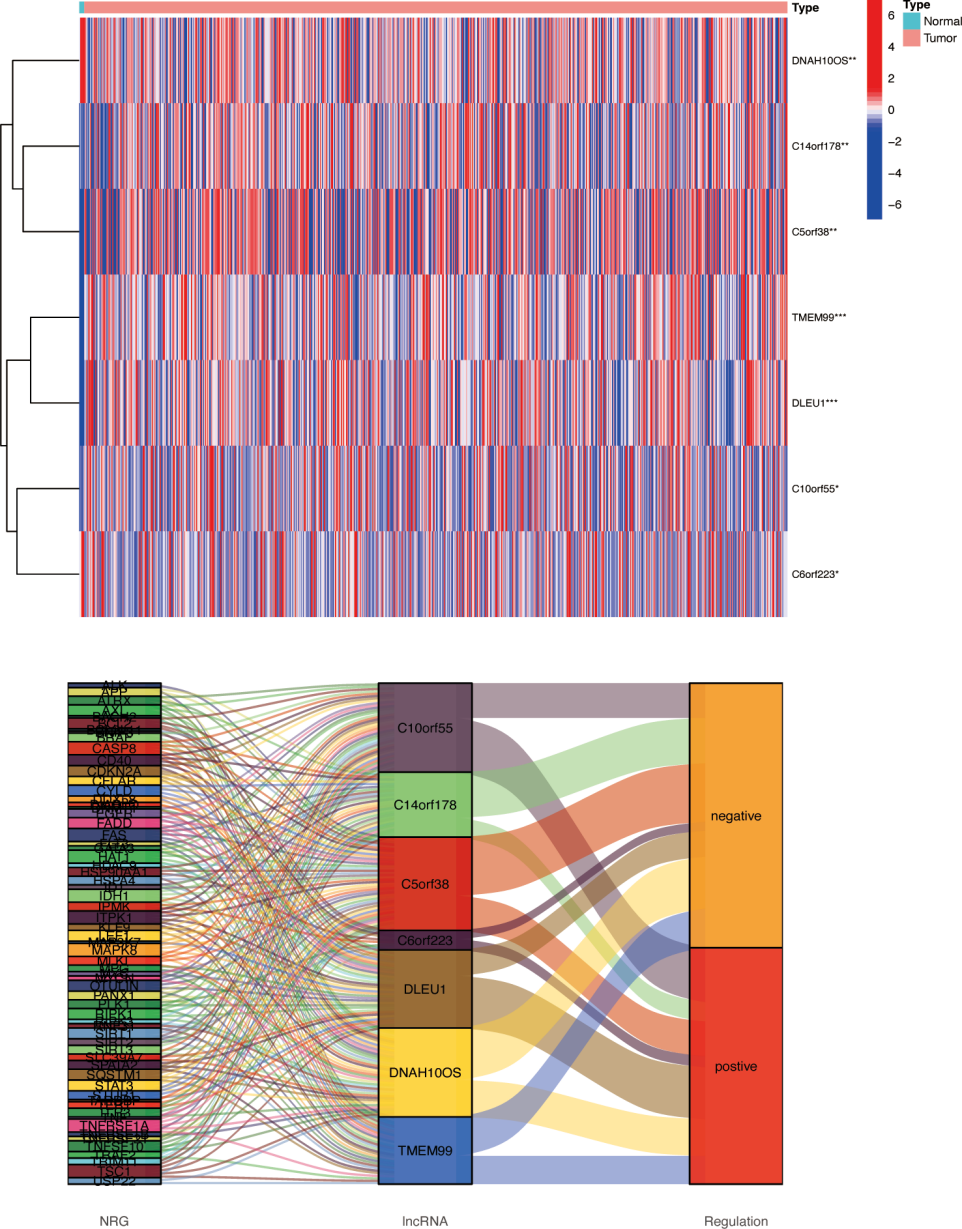
Differences in NRlncRNA expression between normal tissue and tumor tissue. Heat map and Sankey map of differentially expressed NRlncRNA. NRlncRNA, necroptosis-related long non-coding RNA. * if p < 0.05, ** if p < 0.01, and *** if p < 0.001.

Based on univariate Cox regression analysis, seven differentially expressed NRlncRNAs (TMEM99, C10orf55, C6orf223, and DLEU1) had HRs of >1, indicating that they were related to increased risk. The other genes (C14orf178, DNAH10OS, and C5orf38) had HRs of <1 and were protective factors (**Figure 3A**). Subsequently, differential NRlncRNA heat maps and Sankey maps were constructed to observe NRlncRNA expression differences and the regulatory relationship between differential NRlncRNAs and genes, as is shown in **Figure 4**. With the use of the ‘glmnet’ R package, a LASSO-Cox regression model was built to evaluate the prognostic value of these differentially expressed NRlncRNAs. Based on the minimum penalty parameter (λ), six of the seven differentially expressed NRlncRNAs were retained, namely, TMEM99, C10orf55, C6orf223, C14orf178, DNAH10OS, and C5orf38 (**Figures 3B, C**). The prognosis risk score formula was established based on weighted regression coefficients, expression levels, and multivariate Cox regression analysis: risk score = (C14orf178 * −2.47047836968017) + (TMEM99 * 0.890377622191113) + (C10orf55 * 1.92319840764139) + (C6orf223 * 0.584844680876498) + (DNAH10OS * −0.516452244653953) + (C5orf38 * −0.5525866805705). According to the risk score, glioma patients were divided into two groups. The risk score distribution and survival status are shown in **Figures 3G, H**. A survival analysis showed a better prognosis for the low-risk group than for the high-risk group (**Figure 3D**). In the same way, PFS also showed a better prognosis for the low-risk group than for the high-risk group (**Figure 3E**). Meanwhile, the DCA that we constructed showed that when the risk threshold was greater than 6%, the risk model had a good clinical performance (**Figure 3F**). Finally, the promising predictive value for the glioma special model in the whole set was demonstrated by ROC curve analysis (1-year AUC = 0.812, 2-year AUC = 0.840, and 3-year AUC = 0.773) (**Figure 3I**).

### Internal verification, external verification, and model contrast

**Figure 5:**
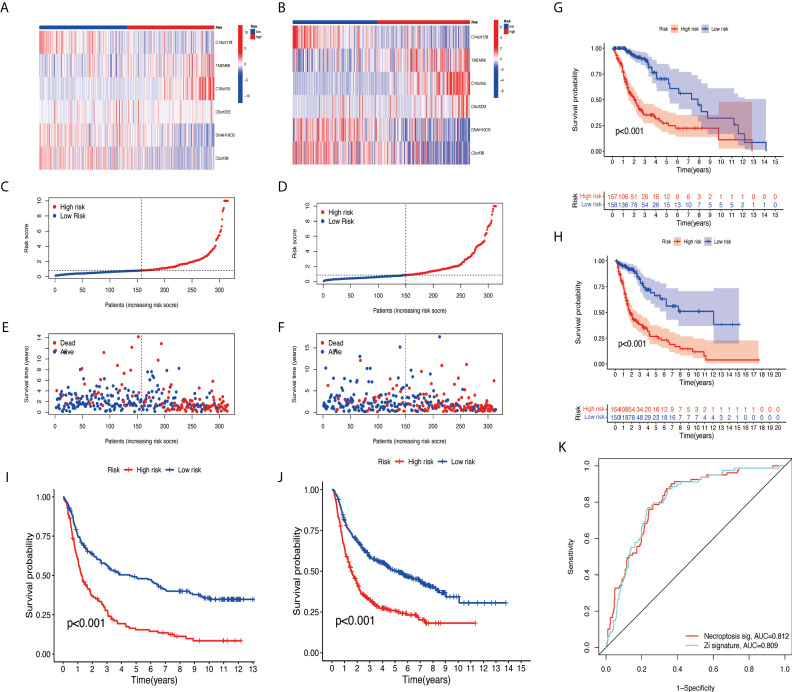
Validation of necroptosis-related lncRNA prognosis signature. **(A, C, E)** The risk score and patients are distributed based on the risk score in the training group. **(B, D, F)** The risk score and patients are distributed based on the testing group’s risk score. **(G)** The survival curves in the training group. **(H)** The survival curves in the testing group. **(I)** The survival curves in CGGA325. **(J)** The survival curves in CGGA693. **(K)** ROC curves between two models. lncRNA, long non-coding RNA; ROC, receiver operating characteristic.

The samples were randomly divided into the training group and testing group at a ratio of 1:1 using the ‘caret’ R package. The training group was used to construct a prognostic model and the testing group to verify it. With the use of the median risk score in the training and testing groups, the risk score for each sample was categorized as high-risk or low-risk. A survival analysis showed a better prognosis for the low-risk group than for the high-risk group (**Figures 5G, H**). The risk curve revealed that the higher the risk score, the lower the patient survival rate (**Figures 5C–F**). According to the heat map, we can see the expression differences of NRlncRNA in different groups (**Figures 5A, B**). Meanwhile, survival analysis was performed using CGGA data (CGGA325 and CGGA693), and the results show that the risk model we constructed also applies to external data (**Figures 5I, J**). The ROC curve we constructed shows that our risk model has certain advantages (**Figure 5K**).

### Cox analysis and prognostic nomogram construction

**Figure 6:**
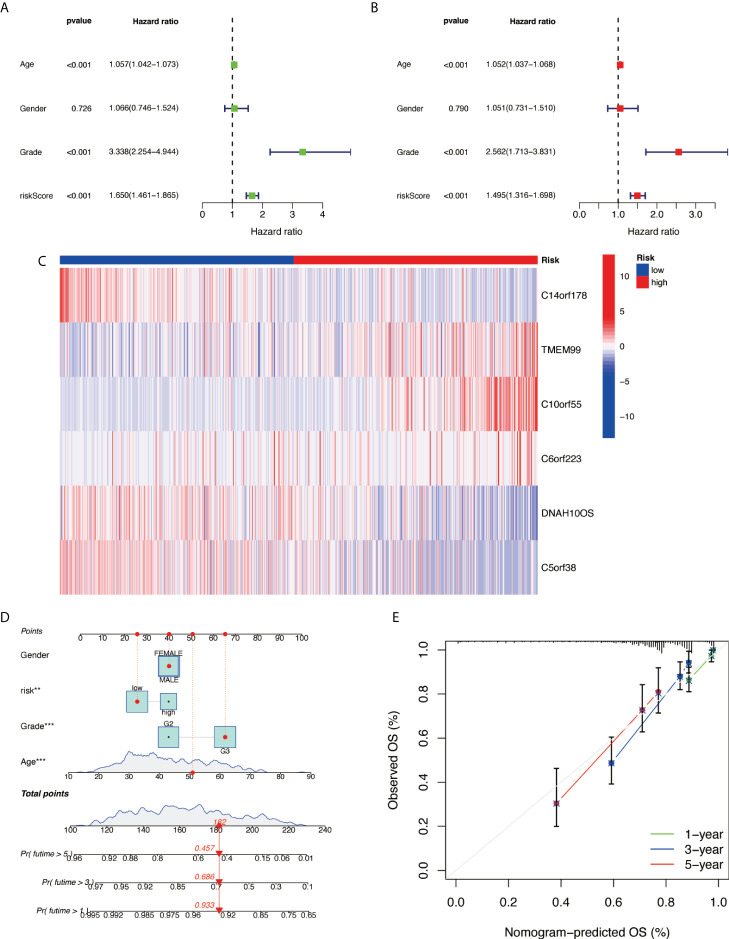
Prognostic nomogram construction. **(A)** Univariate Cox analysis. **(B)** Multivariate Cox analysis. **(C)** Model gene expression heat map between high/low risk. **(D)** Nomograph plot of predicted 1-, 3-, and 5-year overall survival probability based on prognosis signature. **(E)** Calibration plots of the nomogram for predicting the probability of OS at 1, 3, and 5 years in TCGA. OS, overall survival; TCGA, The Cancer Genome Atlas.

Further examination was performed using univariate Cox analysis (**Figure 6A**) and multivariate Cox analysis (**Figure 6B**) to evaluate the accuracy of the independent prognostic signature. Both groups yielded similar results, suggesting that the prognostic signature was effective. To provide the clinician with a quantitative method for predicting the probability of 1-, 3-, and 5-year OS in glioma, a nomogram incorporating risk score, age, gender, and grade as variables was constructed in whole sets (**Figure 6C**). The calibration plots and the predictive curves were close to the ideal curves and showed that the performance of the nomogram was the best in predicting the 1-, 3-, and 5-year OS in whole sets (**Figures 6D, E**). The nomogram can be used to predict the OS rate of different patients according to their conditions to improve the prediction efficiency and accuracy.

### Gene ontology and kyoto encyclopedia of genes and genomes analysis

**Figure 7:**
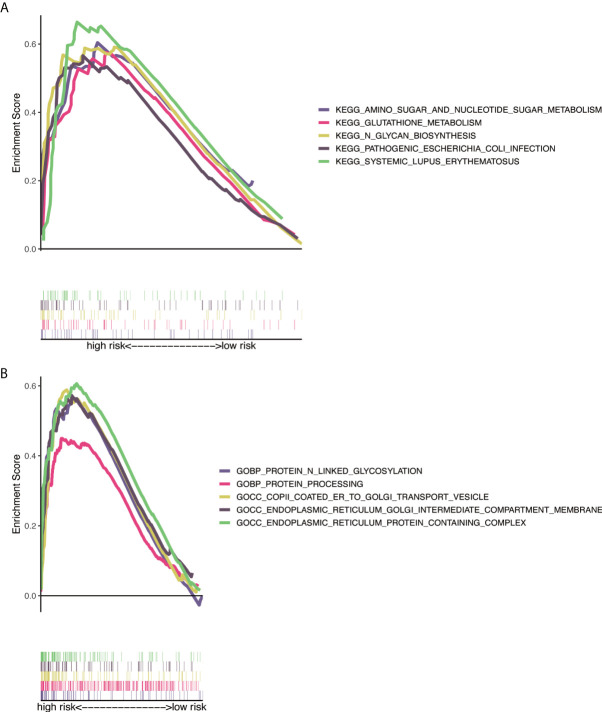
Functional and enrichment pathways analysis. **(A)** GO foundation enrichment analysis in the high-risk group. **(B)** KEGG pathway enrichment analysis in the high-risk group. GO, Gene Ontology; KEGG, Kyoto Encyclopedia of Genes and Genomes.

GO analysis indicated that NRlncRNAs correlated with protein processing and N-linked glycosylation (**Figure 7A**). The KEGG enrichment analysis results are also displayed (**Figure 7B**), including N-glycan biosynthesis, proteasome, amino sugar, and nucleotide sugar metabolism. This result showed that these NRlncRNAs are closely related to protein processing, amino sugar, and nucleotide sugar metabolism, and they play essential roles in the occurrence and development of tumors.

### Immune correlation analysis

**Figure 8:**
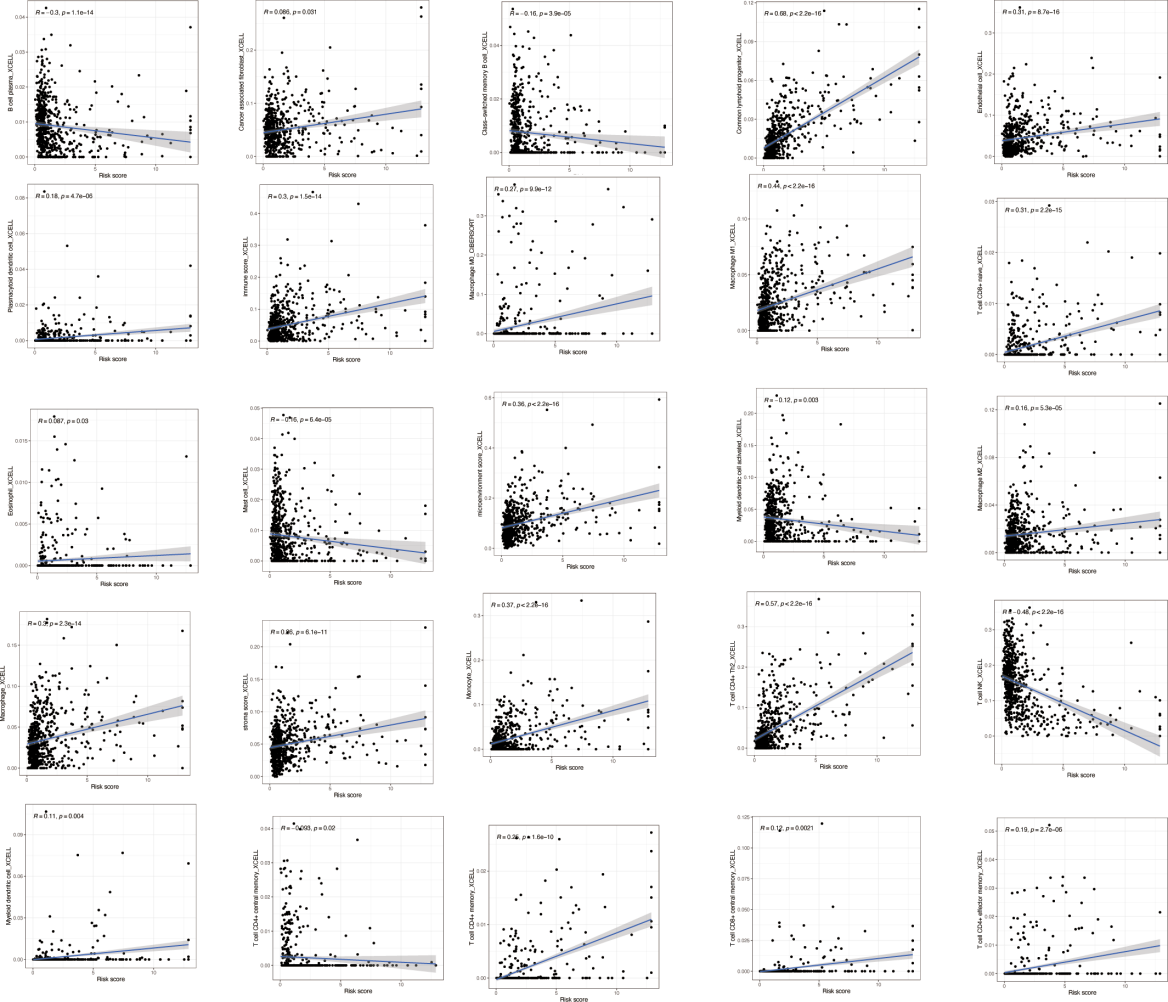
Immune correlation analysis based on NRlncRNAs. Various immune cells are involved in NRlncRNA expression. NRlncRNAs, necroptosis-related long non-coding RNAs.

To explore the immune correlates of risk scores, we explored and validated them by using XCELL. The results showed that a variety of immune cells were positively associated with a patient’s risk score, including B-cell plasma (*R* = −0.3, *p* = 1.1e−14), cancer-associated fibroblast (*R* = 0.086, *p* = 0.031), class-switched memory B cell (*R* = −0.16, *p* = 3.9e−05), common lymphoid progenitor (*R* = 0.68, *p* < 22.2e−16), endothelial cell (*R* = 0.31, *p* = 8.7e−16), plasmacytoid dendritic cell (*R* = 0.18, *p* = 4.7e−06), immune score (*R* = 0.3, *p* = 1.5e−14), macrophage M0 (*R* = 0.27, *p* = 9.9e−12), macrophage M1 (*R* = 0.44, *p* < 2.2e−16), T-cell CD8+ naive (*R* = 0.31, *p* = 2.2e−15), eosinophil (*R* = 0.087, *p* = 0.03), mast cell (*R* = −0.16, *p* = 6.4e−05), microenvironment score (*R* = 0.36, *p* < 2.2e−16), myeloid dendritic cell activated (*R* = −0.12, *p* = 0.003), macrophage M2 (*R* = 0.16, *p* = 5.3e−05), macrophage (*R* = 0.3, *p* = 2.3e−14), stroma score (*R* = 0.26, *p* = 6.1e−11), monocyte (*R* = 0.37, *p* < 2.2e−16), T-cell CD4+ Th2 (*R* = 0.57, *p* < 2.2e−16), T-cell NK (*R* = −0.48, *p* < 2.2e−16), myeloid dendritic cell (*R* = 0.11, *p* = 0.004), T-cell CD4+ central memory (*R* = −0.093, *p* = 0.02), T-cell CD4+ memory (*R* = 0.25, *p* = 1.6e−10), T-cell CD8+ central memory (*R* = 0.12, *p* = 0.0021), and T-cell CD4+ effector memory (*R* = 0.19, *p* = 2.7e−06) (**Figure 8**).

### Immune pathways and underlying mechanisms

**Figure 9:**
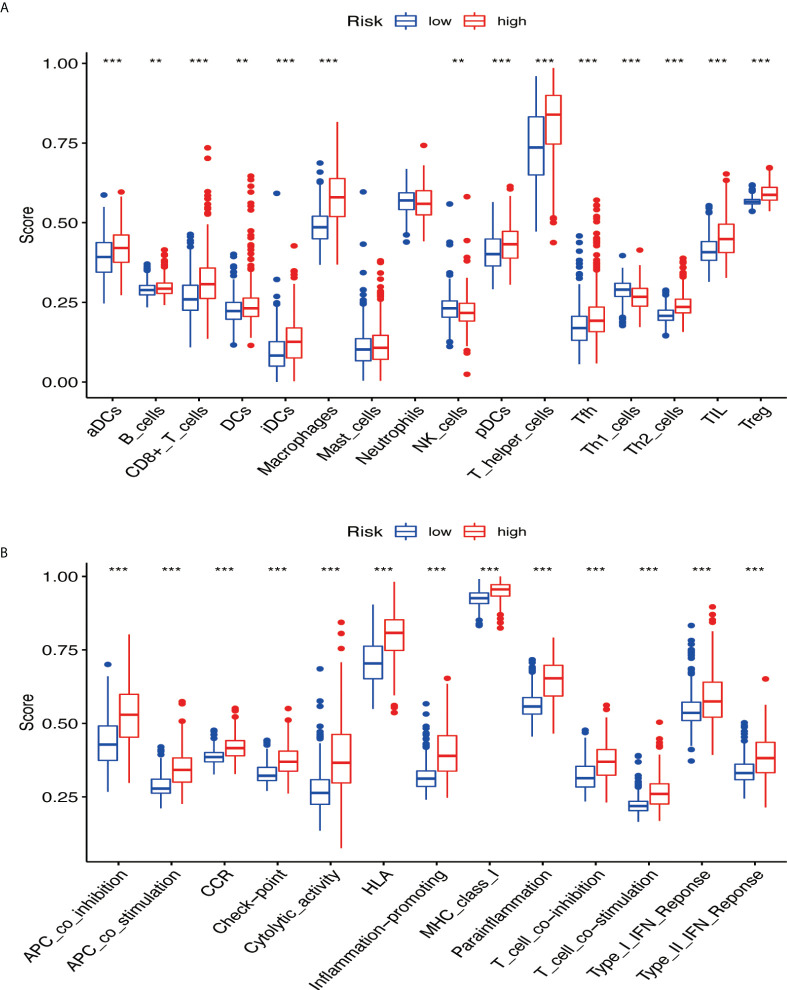
The immune pathways and underlying mechanisms. **(A)** Two immune cells were not related to patient risk score, three immune cells showed a higher degree of infiltration in the low-risk group, and 11 immune cells showed a higher degree of infiltration in the high-risk group. **(B)** Thirteen immune functions were identified. ** if p < 0.01, and *** if p < 0.001.

GSVA was performed to explore the immune pathways and underlying mechanisms related to the glioma patient. A total of 16 differentially enriched immune cells, including two immune cells, were unrelated to the patient risk score. Three immune cells showed a higher degree of infiltration in the low-risk group, and 11 immune cells showed a higher degree of infiltration in the high-risk group (**Figure 9A**). Furthermore, 13 immune functions were identified, including APC co-inhibition, APC co-stimulation, CCR, checkpoint, cytolytic activity, HLA, inflammation promoting, MHC class I, parainflammation, T-cell co-inhibition, T-cell co-stimulation, type I IFN response, and type II IFN response (**Figure 9B**).

### Tumor immune microenvironment and immune checkpoint

**Figure 10:**
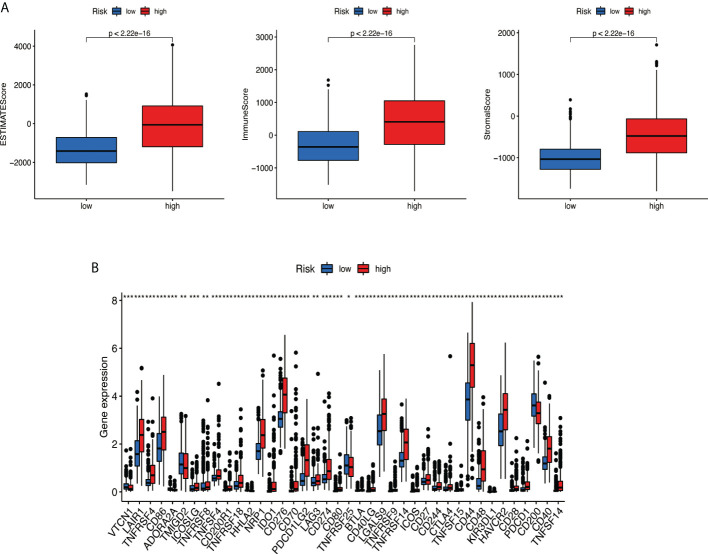
Analysis of tumor microenvironment. **(A)** Stromal cells and immune cells had a higher degree of infiltration in the high-risk group. **(B)** Associated immune gene checkpoints. * if p < 0.05, ** if p < 0.01, and *** if p < 0.001.

Based on the ESTIMATE algorithm, the score of 629 TME clinical samples was calculated. Then, the differences in the score of TME between the high-risk score group and the low-risk score group were compared. It was found that stromal cells and immune cells had a higher degree of infiltration in the high-risk group (**Figure 10A**, all *p* < 0.05). Differential analysis of immune gene checkpoints was performed using R software. The boxplot was created to visualize the amount of gene expression and validated (**Figure 10B**).

### Prediction of potential chemical drugs between two groups

**Figure 11:**
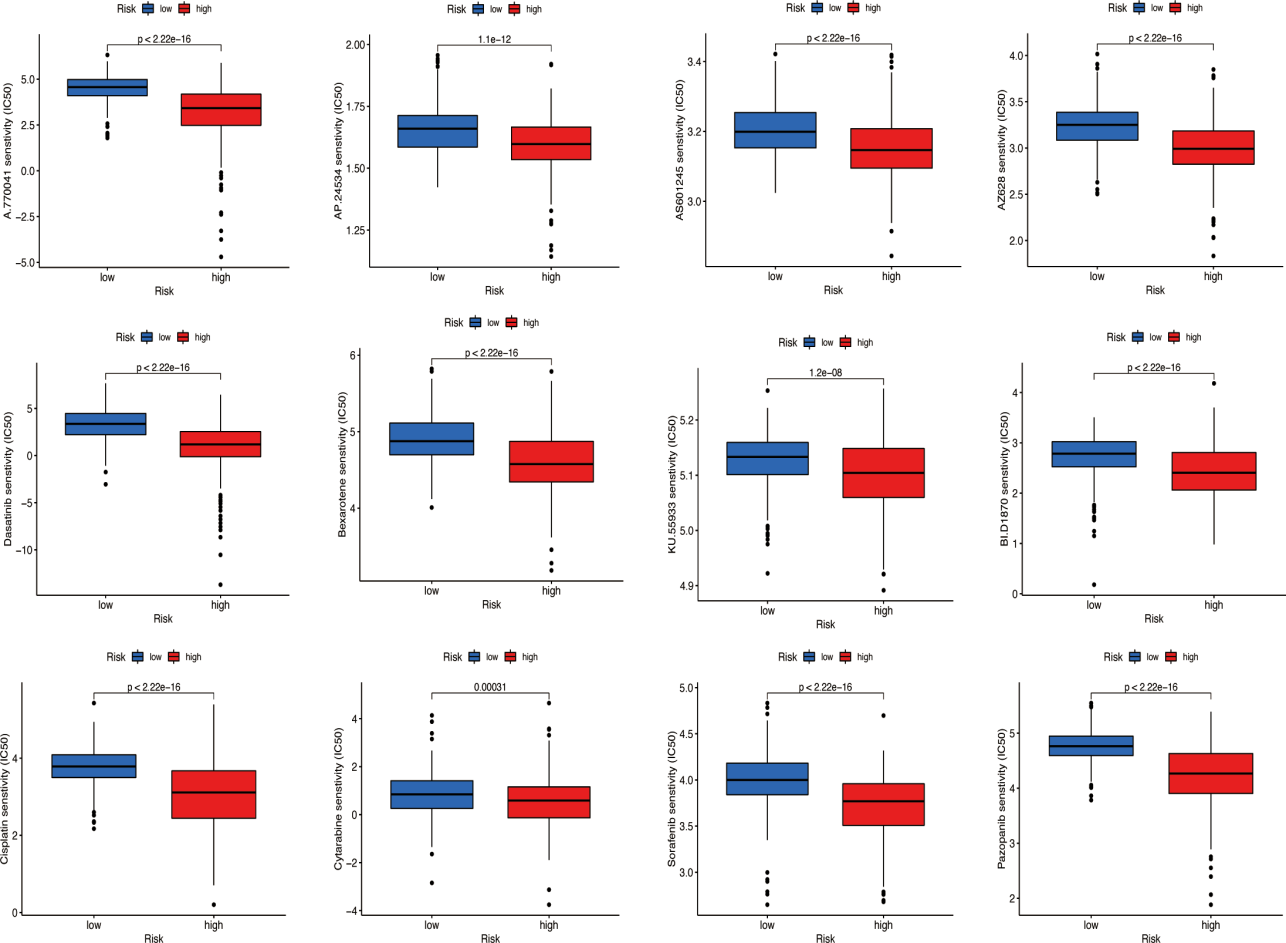
Prediction of potential chemical drugs. These drugs all had higher IC50 in low-risk patients.

According to the ‘prophetic’ algorithm, we predicted the IC50 of 12 common chemotherapeutic agents (A.770041, AP.24534, AS601245, AZ628, parthenolide, dasatinib, bexarotene, KU.55933, BI.D1870, cisplatin, cytarabine, sorafenib, and pazopanib) in high- and low-risk patients and found that these drugs all had higher IC50 in low-risk patients (all *p* < 0.05; **Figure 11**).

### Consensus cluster analysis of prognosis necroptosis-related lncRNAs

**Figure 12:**
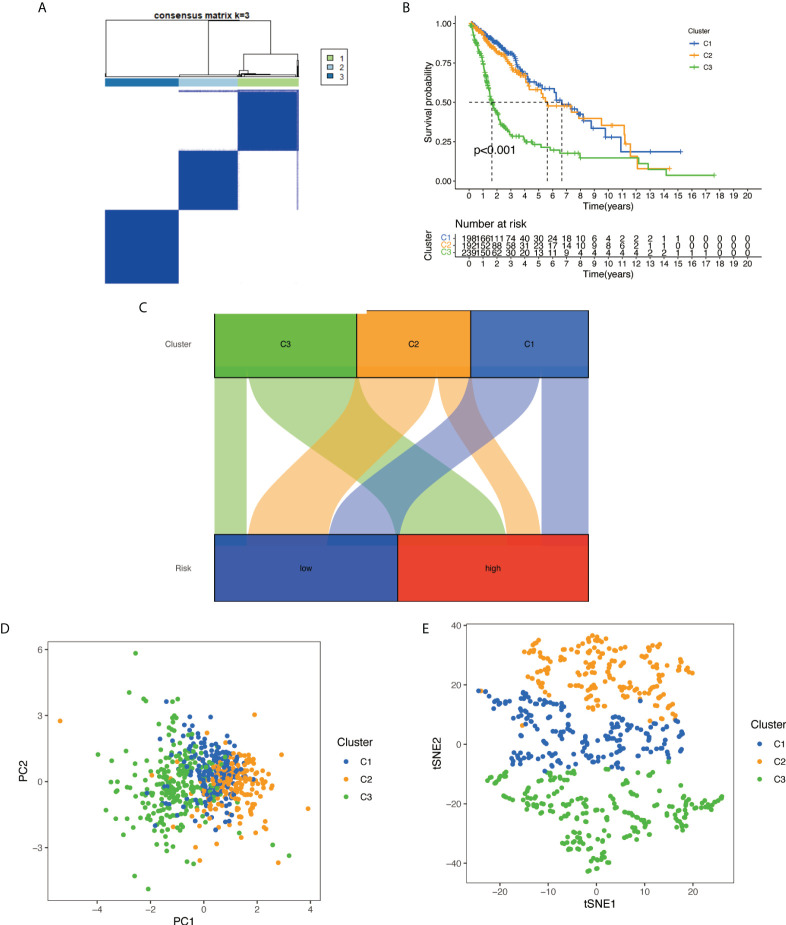
Based on the expression level of necroptosis-related lncRNA, the samples were grouped into subtypes, and survival analysis was conducted: **(A)** glioma patients were divided into three clusters in TCGA. **(B)** Overall survival curve of three clusters in TCGA. **(C)** A Sankey diagram for typing to check the corresponding relationship between typing and risk groups. **(D)** PCA indicated that three subclasses were obtained in TCGA. **(E)** t-SNE indicated that three subclasses were obtained in TCGA. lncRNA, long non-coding RNA; TCGA, The Cancer Genome Atlas; PCA, principal component analysis; t-SNE, t-distributed stochastic neighbor embedding.

With the use of six prognostic necroptosis-related lncRNAs as a marker, the expression levels of each gene in the glioma subtype were examined. All 629 samples in TCGA cohort were subjected to a consensus clustering analysis. The tumor samples were divided into clusters *via* the ‘ConsensusClusterPlus’ package. From two to six, the clustering variable (k) was increased, and it was found that k = 3 had the highest intragroup correlations and the lowest intergroup correlations (**Figure 12A**). The survival analysis revealed remarkable differences between the three subgroups, with Cluster 3 having a worse survival rate than Clusters 1 and 2 (**Figure 12B**). At the same time, a Sankey diagram for typing was built to check the corresponding relationship between typing and risk groups, and the results are shown in **Figure 12C**. PCA and t-SNE revealed there were distinct features between three clusters in each TCGA (**Figures 12D, E**) dataset.

### Cluster immune correlation analysis and drug prediction

**Figure 13:**
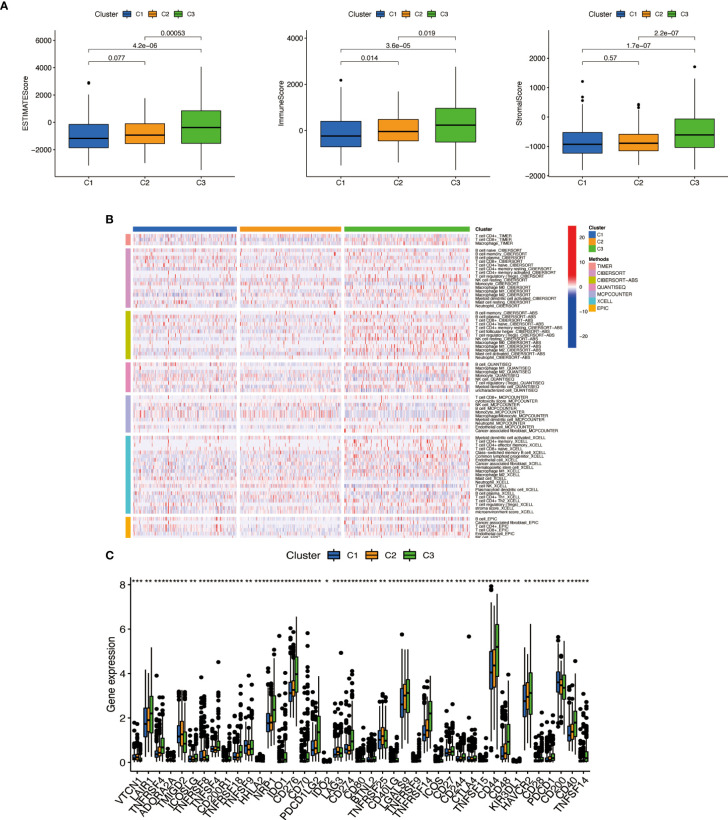
Cluster immune correlation analysis. **(A)** Stromal cells and immune cells had a higher degree of infiltration in Cluster 3. **(B)** An immune-related heat map shows more infiltration of immune cells at Cluster 3. **(C)** The result of immune gene checkpoints. * if p < 0.05, ** if p < 0.01, and *** if p < 0.001.

**Figure 14:**
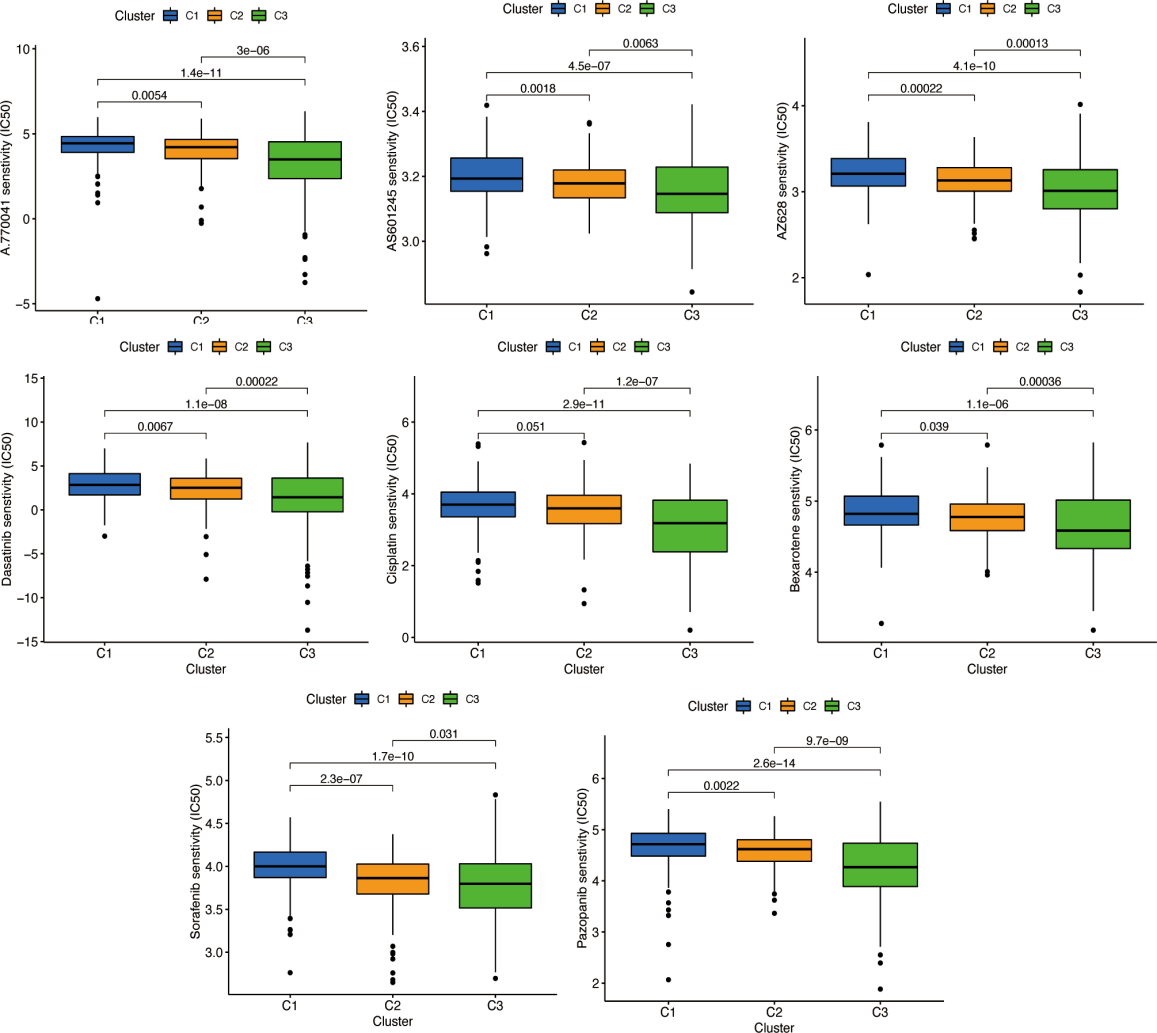
Prediction of potential chemical drugs. These drugs all had higher IC50 in Cluster 3.

We used the same method to perform microenvironment analysis, immune correlation analysis, immune checkpoint identification, and potential drug prediction for tumor subtypes. The results showed that stromal cells and immune cells had higher infiltration in Cluster 3 (**Figure 13A**). We constructed an immune-related heat map showing more infiltration of immune cells at Cluster 3 (**Figure 13B**). The result of immune gene checkpoints is shown in **Figure 13C**. Drug prediction showed that these drugs (A.770041, AS601245, AZ628, dasatinib, bexarotene, cisplatin, sorafenib, and pazopanib) all had lower IC50 in Cluster 3; there were four fewer drugs compared to the risk group (**Figure 14**).

## Discussion

Glioma, especially glioblastoma, causes the most destruction in the human nervous system. Over the past decade, tremendous advances have been made in diagnosing and treating tumors; gliomas quickly proliferate, making them difficult to cure and prone to recurrence after surgery, leading to poor clinical prognosis for glioma patients (14). To provide accurate prognoses for gliomas, it is imperative to investigate the early detection of gliomas and accurately predict their outcome.

Nowadays, lncRNAs, especially necroptosis-related lncRNAs, are gaining more attention from researchers. Multiple signaling and control functions have been proven to exist in lncRNAs, which may affect multiple aspects of tumor development (15). Moreover, researchers have discovered that the immune microenvironment influences tumor growth (16, 17). The upregulation of various lncRNAs by gliomas has been confirmed, and these lncRNAs contribute to the proliferation and invasion of glioma cells, such as CRNDE and H19 (18, 19). Furthermore, some lncRNAs are downregulated in glioma, which may possess similar properties to tumor suppressor genes and inhibit tumor cell proliferation, promoting apoptosis, such as WDR11 and MEG3 (20, 21).

In this study, eight necroptosis-related lncRNAs were particularly associated with glioma. In LASSO analysis, six necroptosis-related lncRNAs (TMEM99, C10orf55, C6orf223, C14orf178, DNAH10OS, and C5orf38) were retained in our RS formula. The RS formula could effectively predict the prognosis of glioma patients (1-year AUC = 0.812, 2-year AUC = 0.840, and 3-year AUC = 0.773). This shows the success of the model building.

In addition, few similar studies have used necroptosis-related lncRNAs to predict glioma-related genes. Our results provide additional reliable evidence to support further research.

## Conclusion

In conclusion, we verified that necroptosis-related lncRNAs play a significant role in the occurrence and development of glioma. The results of these studies added some valuable guidance to understanding glioma pathogenesis and treatment, and these necroptosis-related lncRNAs may be used as biomarkers and therapeutic targets for glioma prevention.

## Data availability statement

The datasets presented in this study can be found in online repositories. The names of the repository/repositories and accession number(s) can be found in the article/supplementary material.

## Author contributions

MG designed the study. DC, JY, and BH acquired and analyzed the data. LK, BX, CD, HL, YZ, SY, FW, and YL contributed analysis tools. DC wrote the paper. All authors contributed to the article and approved the submitted version.

## Funding

This study was supported by the National Natural Science Foundation of China (Nos. 82173384 and 81773161).

## Conflict of interest

The authors declare that the research was conducted in the absence of any commercial or financial relationships that could be construed as a potential conflict of interest.

The reviewer GY declared a shared parent affiliation with the authors to the handling editor at the time of review.

## Publisher’s note

All claims expressed in this article are solely those of the authors and do not necessarily represent those of their affiliated organizations, or those of the publisher, the editors and the reviewers. Any product that may be evaluated in this article, or claim that may be made by its manufacturer, is not guaranteed or endorsed by the publisher.
